# Bile acids and their receptors: modulators and therapeutic targets in liver inflammation

**DOI:** 10.1007/s00281-022-00935-7

**Published:** 2022-04-12

**Authors:** Anna Bertolini, Romina Fiorotto, Mario Strazzabosco

**Affiliations:** 1 Section of Digestive Diseases, Yale Liver Center, Yale School of Medicine, PO Box 208019, New Haven, CT 06520-8019 USA; 2 Department of Pediatrics, Section of Molecular Metabolism and Nutrition, University Medical Center Groningen, Groningen, The Netherlands

**Keywords:** Bile acids, Bile acid receptors, Inflammation, Liver, Cholestasis, FXR, TGR5

## Abstract

Bile acids participate in the intestinal emulsion, digestion, and absorption of lipids and fat-soluble vitamins. When present in high concentrations, as in cholestatic liver diseases, bile acids can damage cells and cause inflammation. After the discovery of bile acids receptors about two decades ago, bile acids are considered signaling molecules. Besides regulating bile acid, xenobiotic, and nutrient metabolism, bile acids and their receptors have shown immunomodulatory properties and have been proposed as therapeutic targets for inflammatory diseases of the liver. This review focuses on bile acid–related signaling pathways that affect inflammation in the liver and provides an overview of the preclinical and clinical applications of modulators of these pathways for the treatment of cholestatic and autoimmune liver diseases.

In humans, the primary bile acids cholic acid (CA) and chenodeoxycholic acid (CDCA) are synthesized in the liver from cholesterol and secreted as bile components into the duodenum. In the small intestine, bile acids aid in the absorption of fat, cholesterol, and fat-soluble vitamins and orchestrate bile acid, lipid, and energy metabolism by acting as ligands for bile acid receptors. The intestinal microbiota transforms the primary bile acids CA and CDCA into the secondary bile acids deoxycholic acid (DCA) and lithocholic acid (LCA), respectively. In mice, CDCA can also be converted to muricholic acid (MCA), which renders mouse bile more hydrophilic than human bile [1]. A small amount (~ 5%) of bile acids are lost in feces, whereas the remainder is reabsorbed either actively in the ileum (conjugated bile acids via the apical sodium–dependent bile acid transporter, ASBT) or passively in the colon (deconjugated bile acids) [2].

Upon reabsorption in the ileum, bile acids bind to the nuclear receptor farnesoid X receptor (FXR), which regulates the expression of genes involved in the uptake and efflux of bile acids to prevent their accumulation, and which cross-signals with other nuclear receptors to regulate bile acid, xenobiotic, and nutrient metabolism. Importantly, bile acid binding to intestinal FXR induces the production of fibroblast growth factor 19 (FGF19), which travels to the liver via the portal circulation along with the reabsorbed bile acids to inhibit hepatic bile acid synthesis, effectively providing a negative feedback mechanism to maintain bile acid pool homeostasis. FXR expressed in the liver further regulates bile acid synthesis and nutrient signaling [3].

Bile acids returning from the portal circulation and bile acids in the systemic circulation are taken up by hepatocyte Na^+^-dependent taurocholate cotransporting peptide (NTCP) and, together with the newly synthesized bile acids, are secreted into canaliculi via the bile salt export pump (BSEP), thereby completing their enterohepatic circulation [4] (Fig. 1).

**Fig. 1:**
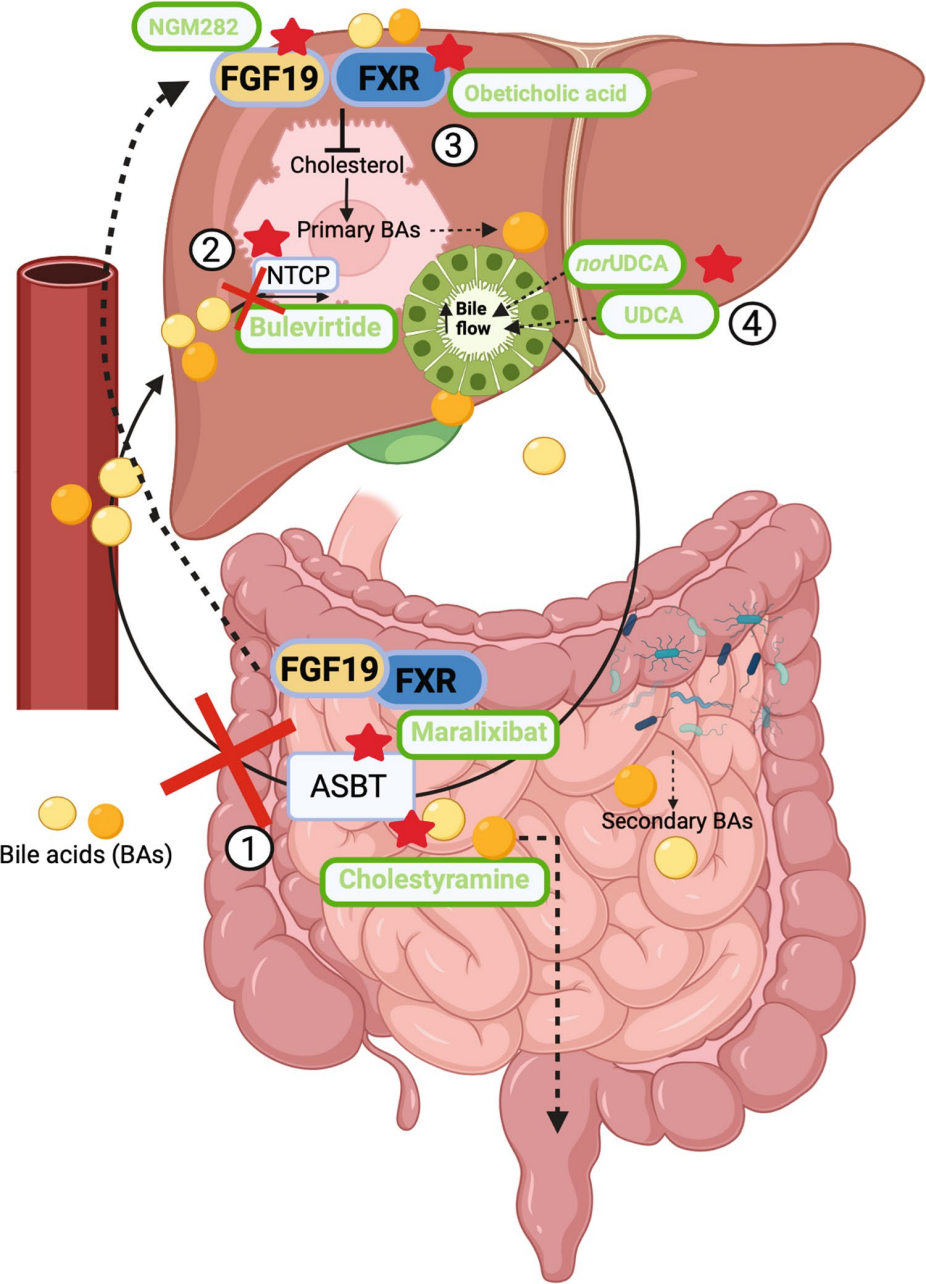
Bile acid homeostasis and bile acid–related treatments in cholestatic liver diseases. Bile acids are synthesized by hepatocytes in the liver from cholesterol and secreted into bile. After being modified by the bile duct epithelium, the bile is secreted in the duodenum to accomplish digestive functions. Under the action of the intestinal microbiota, primary bile acids are modified into secondary bile acids. Bile acids are in large part re-absorbed by ileal ASBT to be returned to the liver via the portal circulation. Upon intestinal reabsorption, bile acids activate FXR-FGF19 that negatively regulates bile acids synthesis in the liver. The different steps of the enterohepatic circulation can potentially be targeted in cholestatic liver diseases to antagonize the effects of bile acids accumulation. In the figure are reported selected drugs that are in experimental trial or approved for (1) interrupting the enterohepatic circulation, (2) reducing bile acids uptake, (3) reducing bile acids synthesis, (4) increasing bile flow and decreasing bile acid hydrophobicity. ASBT: apical sodium–dependent bile acid transporter; BAs: bile acids; FXR: farnesoid X receptor; FGF19: fibroblast growth factor 19; nor-UDCA: nor-ursodeoxycholic acid; NTCP: Na^+^-dependent taurocholate cotransporting peptide; UDCA: ursodeoxycholic acid

Besides their well-studied roles in fat absorption and nutrient signaling, bile acids and their receptors contribute to the modulation of immunity. The interplay between bile acids and immunity is multifaceted and includes physicochemical interactions of bile acids with cells and immune-related pathways dependent or independent from interaction with bile acid receptors as well as interactions between bile acids and the gut microbiota. The scope of this review is to provide an overview of the bile acid–related signaling pathways that affect inflammation in the liver, and to review the preclinical and clinical evidence of how modulators of these pathways and receptors perform in the treatment of cholestatic and autoimmune liver diseases.

## Bile acid accumulation and inflammation in cholestasis

In cholestatic diseases, elevated levels of bile acids within the liver cause injury and inflammation, which can progress to fibrosis and cirrhosis. The exact mechanisms of liver injury consequent to bile acid accumulation have not been fully clarified and are likely multifactorial and different in different types of cholestatic diseases. Bile acids are amphipathic molecules with different degrees of hydrophobicity, with ursodeoxycholic acid (UDCA) being the most hydrophilic and CA, CDCA, DCA, and LCA being progressively more hydrophobic [5].

**Fig. 2:**
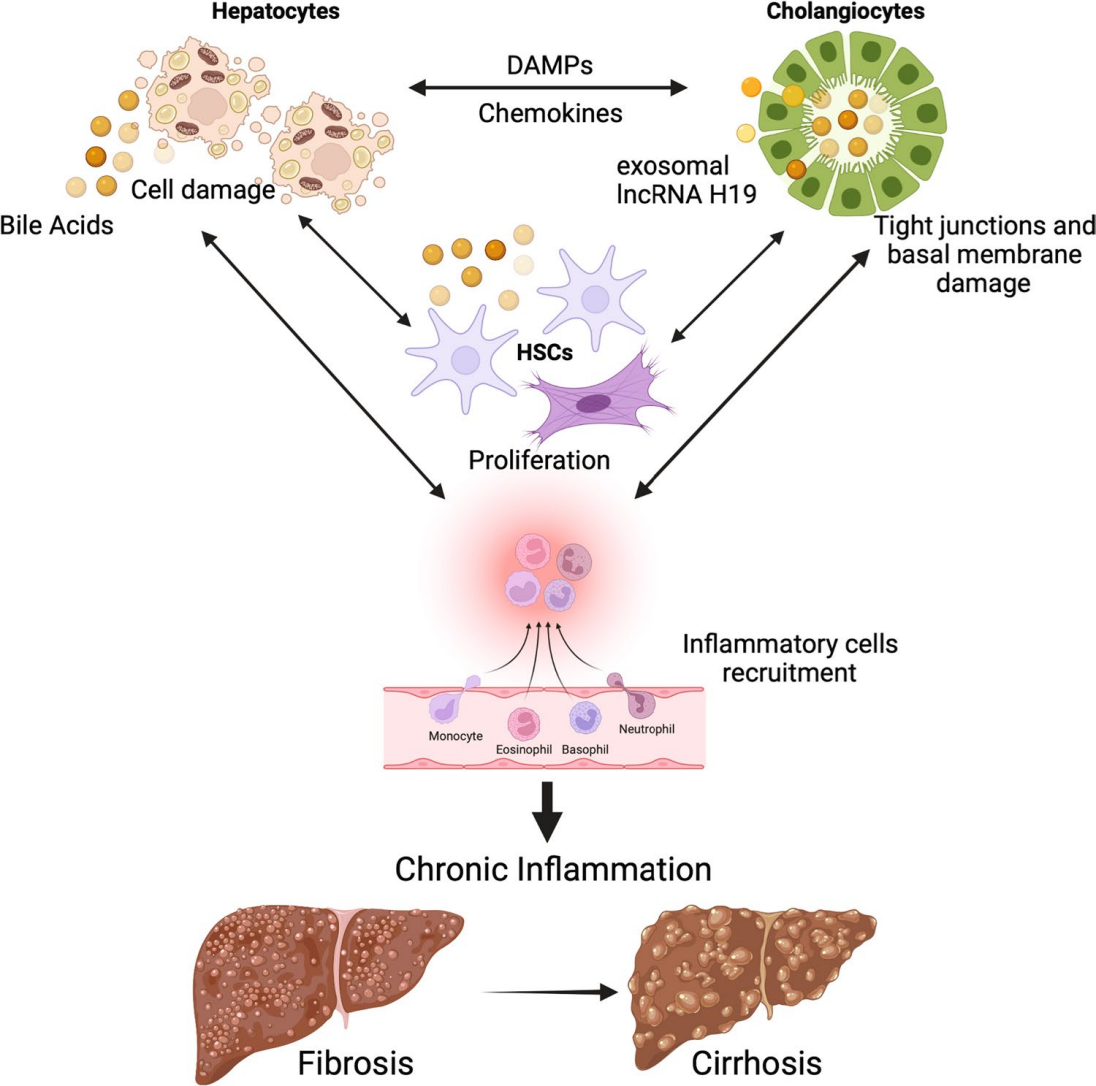
Effects of bile acid accumulation in the liver. Bile acid accumulation in cholestatic conditions can activate different signaling pathways in different cell types of the liver. Damaged hepatocytes can initiate an inflammatory response by secreting chemokines and DAMPs that activate other cells (i.e., cholangiocytes, HSCs, and inflammatory cells). High levels of bile acids can also directly disrupt tight junctions and basal membrane of bile ducts, leading to activation of cholangiocytes and perpetuation of the inflammatory/fibrotic response with proliferation and activation of HSCs. Persistent chronic inflammation and fibrosis can progress into cirrhosis. DAMPs: damage-associated molecular patterns; HSCs: hepatic stellate cells; lncRNA H19: long non-coding RNA H19

Because of their hydrophobicity, based on early in vitro studies, it was proposed that bile acids could directly lyse hepatocyte cell membranes [6–8] or induce hepatocyte apoptosis [9]. However, these studies did not always recapitulate the cholestatic situation in vivo in terms of bile acid species and concentrations used to challenge hepatocytes. The mechanisms mediating bile acid–induced hepatocyte death are still debated [10] and different bile acid concentrations at different stages of cholestasis likely lead to different responses [11]. There is evidence supporting direct bile acid–mediated hepatocyte death, which is a pro-inflammatory event that likely propagates inflammation to other liver cell types. Hepatocyte necroptosis was observed in hepatocytes from PBC patients and after bile duct ligation in mice [12]. Another study suggested that hepatocyte death occurs primarily in the acute phase of cholestasis, 1–3 days after bile duct ligation in mice. In this situation, the pathogenetic sequence sees rupture of the hepatocyte apical membrane, entry of bile, and death of single cells followed by the death of surrounding hepatocytes (bile infarcts). This response was not observed in chronic cholestasis, either after 3 days from bile duct ligation or in *Mdr2*^*−/−*^ mice, which chronically lack phospholipid secretion in bile [13]. Recent research suggests that increased bile acid levels in cholestatic conditions induce the secretion of cytokines by hepatocytes [14–17], which can recruit neutrophils to initiate the inflammatory response [16, 18] (Fig. 2). In these studies, bile acid–induced secretion of cytokines by hepatocytes was unrelated to cellular toxicity, apoptosis, or necrosis [14, 15], required bile acid uptake by hepatocytes via the bile acid importer NTCP [16], and was mediated via the nuclear factor of activated T-cells (NFAT) and toll-like receptor 9 (TLR9) [16, 17]. Consistently, NTCP deficiency, which prevents bile acid uptake by hepatocytes, does neither cause a hepatic phenotype in humans [19], nor in mice [20]. Instead, deficiency of the bile salt export pump BSEP (causing progressive familial intrahepatic cholestasis type 2, PFIC2) and FIC1 (causing PFIC1), in which bile acids enter hepatocytes but cannot be efficiently secreted into bile, causes severe liver injury [10, 21]. Unlike in hepatocytes, bile acid–induced cytokine secretion was not observed in liver non-parenchymal cells using the same bile acid concentrations [16]. Hepatic stellate cells (HSC) do not express NTCP and do not take up bile acids. Whereas no bile acid–induced apoptosis was observed in HSC, bile acids induced HSC proliferation [22], which promotes fibrosis (Fig. 2).

Central to cholestatic diseases is cholangitis, suggesting either direct or indirect bile acid–related damage to cholangiocytes. Cholangiocytes lining bile ducts are routinely exposed to high concentrations of bile acids in bile without sustaining injury. This can likely be attributed to (1) bile acid micellization with cholesterol and phospholipids in bile; (2) the capability of cholangiocytes to secrete bicarbonate in the lumen [23] that, in the presence of glycoproteins and other mucus-like components, may form a glycocalyx on the cholangiocyte luminal side; evidence so far is limited to in vitro studies, but this bicarbonate shield (or umbrella) may protect against bile acid–induced injury [24]; (3) the expression of FXR and its target genes that may be orchestrated to prevent intracellular bile acid accumulation during cholestasis [25]. It was proposed that free biliary bile acids (i.e., not micellized) can damage cholangiocyte membranes [26]. PFIC3, caused by deficiency of multidrug resistance protein 3 (MDR3), a transporter that facilitates phospholipids secretion in the canaliculus [21], is modelled by *Mdr2*^*−/−*^ mice, which feature sclerosing cholangitis. In *Mdr2*^*−/−*^ mice, however, cholangiocyte death is a late pathogenic event, suggesting it may not be caused by bile acid cytotoxicity. This study suggested that bile acids damage cholangiocyte tight junctions and basement membranes first, leading to bile leakage in the periductal area, which initiates the inflammatory and fibrotic response (Fig. 2). Cholangiocyte death would occur after the insurgence of fibrosis, which may deprive cholangiocytes from their blood supply [27]. In bile duct–ligated mice, cholangiocyte proliferation and periportal fibrosis occur after hepatocyte death [12]. Interestingly, cholangiocytes are capable of secreting inflammatory mediators to induce neutrophil activation in response to stimuli such as pathogen-associated molecular patterns (PAMPs) [28–33]. Whether induction of cytokine and chemokine expression occurs in cholangiocytes directly in response to bile acids in cholestatic conditions is still debated due to conflicting results [16, 34]. A series of studies showed that TCA stimulated cholangiocyte proliferation [35] and that cholangiocyte expression of exosomal lncRNA H19 in response to cholestatic injury promoted HSC activation and proliferation [36], as well as macrophage activation [37] to increase cholestatic liver injury (Fig. 2). Cholangiocyte mitochondrial damage was observed in isolated bile duct units in response to unconjugated, but not conjugated bile acids [38]. However, bile contains almost exclusively conjugated bile acids [39], and the authors did not observe cholangiocyte damage when perfusing isolated rat livers with unconjugated bile acids [38]. Patients with rare mutations impairing bile acid conjugation show signs of biliary ductular reaction and cholangiopathy, although inconsistently [27].

In summary, there is evidence supporting direct cytotoxic effects of bile acids on hepatocytes, especially in the acute phase of cholestatic injury, whereas damage to cholangiocytes seems primarily directed at the tight junction and basement membrane. Hepatocyte death likely initiates an inflammatory response that affects other liver cell types, which in turn further amplify inflammation. Independently from cell death, there is evidence supporting a role for bile acids in the induction of pro-inflammatory responses in hepatocytes, as well as in the proliferation of cholangiocytes and HSC. Interestingly, different signaling pathways were described in different cell types, highlighting the multifactoriality of the response to bile acid overload that ultimately results in cholestatic liver injury (Fig. 2).

## Bile acid receptors and inflammation in cholestatic and autoimmune liver diseases

Immune modulation by bile acid has long been hypothesized. Early studies investigated whether elevated serum bile acids in cholestatic liver diseases could be responsible for infectious complications and endotoxemia by directly suppressing the immune response. Accordingly, numerous in vitro studies were carried out to assess the effect of bile acids on immune cell function. Lymphocyte proliferation, immunoglobulin production, and cytokine secretion were suppressed by bile acids [40–44]. Decreased cytokine release by monocytes upon bile acid stimulation was reported by several [43, 45, 46], but not all [47] studies. The phagocytic function of the Kupffer cell was also reported to be decreased by bile acids [48, 49].

On the other hand, a recent study found that bile acids act as damage-associated molecular patterns (DAMPs) that can activate the NOD-, LRR- and pyrin domain–containing protein 3 (NLRP3) inflammasome in macrophages by promoting intracellular calcium influx. In this study, the promotion of inflammasome activation by bile acids was synergistic with LPS and, in vivo, cholestasis aggravated LPS-induced sepsis [50].

The mechanisms underlying the immune-modulating effects of bile acids were studied only recently, after it was discovered that most of the biological actions of bile acid are mediated through the modulation of bile acid receptors. In the next sections, we review the role of bile acid receptors (FXR, TGR5, and PXR) in modulating inflammation and the preclinical and clinical evidence assessing their utility in the treatment of liver diseases.

### FXR

Farnesoid X receptor (FXR, NR1H4) is a nuclear receptor central to nutritional homeostasis. Its major endogenous ligands are bile acids, with CDCA > DCA > LCA > CA in order of FXR activation potency [51, 52]. The mouse bile acid muricholic acid (MCA), which is derived from CDCA by the enzyme Cyp2c70, is a FXR antagonist [53]. The use of *Cyp2c70*^*−/−*^ mice with a human-like bile acid pool is thus recommended in preclinical studies testing FXR modulators [1]. Of note, being a nuclear receptor, FXR activation requires cellular entry of bile acids. In response to ligand activation, FXR regulates the fed state response by modulating the expression of genes involved in (a) bile acid homeostasis (to maintain the bile acid pool size by regulating the amount of newly synthesized bile acids), (b) glucose homeostasis (to reduce postprandial glycemia by limiting hepatic glucose generation), and (c) lipid metabolism (to reduce hepatic fatty acid generation, storage, and release) [54]. A favorable effect of FXR agonism in liver diseases is thought to arise from the reduction of bile acid synthesis and accumulation and increase in bile acid and xenobiotic modification and secretion as well as decrease in hepatic lipogenesis [55]. Besides the well-known role as nutritional homeostat, FXR is also involved in the inflammatory response, which is the focus of this section. As for tissue distribution, the highest mRNA expression of NR1H4 is found in the liver [56, 57]. Here, NR1H4 is mostly expressed by cholangiocytes and hepatocytes and, to a lower extent, by Ito cells, Kupffer cells, and T cells. Other cell types that highly express NR1H4 are enterocytes in the intestine and cells lining the collecting duct system in the kidney [56, 57]. Importantly, in the mouse and under physiological conditions, FXR seems to be basally active in the intestine, but not in the liver. However, in the liver, FXR becomes strongly activated under cholestatic conditions [58].

#### FXR and inflammation

As for other nuclear receptors involved in nutrient metabolism [59], the expression and activation of FXR are repressed during inflammation [60–63]. Since FXR activation is generally anti-inflammatory, FXR repression during inflammation could allow for the inflammatory response to be amplified.

**Fig. 3:**
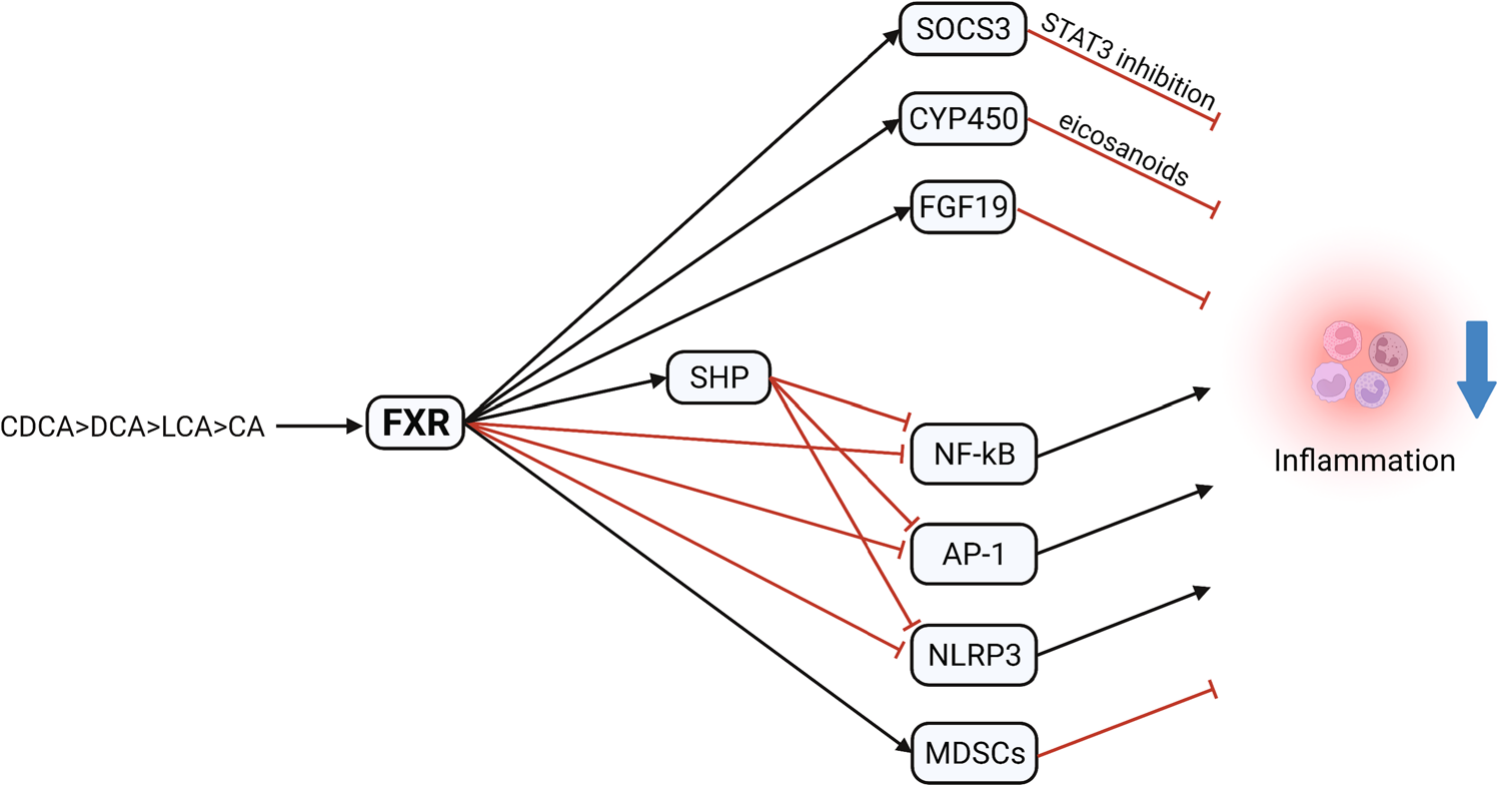
Effect of FXR activation on liver inflammation. Activation arrows are indicated in black and inhibition arrows in red. AP-1: activator protein 1; CA: cholic acid; CDCA: chenodeoxycholic acid; CYP450: cytochrome P450 family 7 subfamily A member 1; DCA: deoxycholic acid; FGF19: fibroblast growth factor 19; FXR: farnesoid X receptor; LCA: lithocholic acid; MDSCs: myeloid-derived suppressor cells; NF-κB: nuclear factor kappa-light-chain-enhancer of activated B cells; NLRP3: NOD-, LRR- and pyrin domain–containing protein 3; SHP: small heterodimer partner; SOCS3: suppressor of cytokine signaling 3

Several members of the nuclear receptor superfamily, including FXR, repress pro-inflammatory genes by regulating the transcription factors that control the expression of these genes, mainly NF-κB and AP-1, in a process known as transrepression [64]. NF-κB is a family of transcription factors that regulates the transcription of an array of pro-inflammatory genes [65]. FXR activation was shown to antagonize NF-κB activation in vitro in hepatocytes, macrophages, enterocytes, other cell types, and in vivo in liver tissue [63, 66–70]. NF-κB inhibition by FXR can also occur via small heterodimer partner (SHP) activation [67]. SHP, an atypical nuclear receptor and a FXR target gene, also prevents AP-1 binding to inflammatory genes [71] and downregulates the expression of the chemokine CCL2 [72]. Furthermore, in cholestasis, loss of SHP was linked to increased lncRNA H19 [73], which is pro-inflammatory. As another mechanism, in a study in hepatocytes, the inhibition of NF-κB signaling by the FXR agonist obeticholic acid (OCA) was dependent on the induction of cytochrome P450 epoxygenases, the enzymes responsible for the synthesis of anti-inflammatory eicosanoids [70]. As discussed in the previous section, hepatocytes responded to bile acids with *induction* of cytokines. These effects were observed in absence of NF-κB stimulation and were FXR-independent [14, 16]. Interestingly, post-translational modifications of FXR can affect the signaling pathways it modulates. FXR sumoylation is promoted by FXR agonism. SUMOylated FXR transrepresses NF-κB signaling without affecting classical FXR target genes such as SHP. On the contrary, FXR acetylation, which is constitutively active in obesity, promotes hepatic inflammation by inhibiting FXR sumoylation [74]. An FXR modulator that represses inflammation via NF-κB without inducing other classical FXR target genes was developed, demonstrating that gene-selective FXR modulation is possible [75]. FXR activation thus decreases inflammation by repressing NF-κB and AP-1 via several mechanisms (Fig. 3).

In addition to repressing NF-κB signaling, FXR affects inflammasome activation. Inflammasomes are multiprotein complexes that control the inflammatory response and are assembled in response to PAMPs and DAMPs [65]. The FXR target gene SHP was shown to repress NLRP3 formation by inhibiting NLRP3 binding to ASC [76]. Additionally, it was observed that FXR could inhibit NLRP3 by directly interacting with NLRP3 and caspase 1 [50]. In mouse models of alcoholic liver disease, FXR agonism increased NLRP3 ubiquitination, which was associated with decreased steatosis and inflammation [77]. Thus, FXR negatively regulates the NLRP3 inflammasome (Fig. 3).

Several studies utilizing mouse models of different liver diseases have assessed the effectiveness of FXR agonism at decreasing hepatic inflammation and shed light on additional anti-inflammatory mechanisms, in addition to negative regulation of NF-κB and NLRP3 (Fig. 3). In mouse models of LPS-induced liver injury, FXR agonism decreased LPS-induced hepatic inflammation [78, 79]. Xu et al. [80] showed that the anti-inflammatory effects of FXR agonism in LPS-induced liver injury were mediated by increased expression of suppressor of cytokine signaling 3 (SOCS3), which downregulates cytokine-STAT3 signaling. Of note, STAT3 signaling is involved in tumorigenesis [81]. Accordingly, ageing *Fxr*^*−/−*^ mice are prone to liver inflammation and spontaneous tumor development [82–85]. FXR is downregulated in hepatocellular carcinoma and cholangiocarcinoma [86, 87], whereas there is experimental evidence suggesting that FXR activation reduces the carcinogenic potential in both types of cancers (the reader is referred to recent, comprehensive reviews on this topic, [88, 89]). *Fxr*^*−/−*^ mice are also more susceptible to autoimmune hepatitis induced by concanavalin A (Con A) and FXR agonism in wild-type mice attenuated liver damage. It was found that FXR in NKT cells activates SHP-mediated inhibition of osteopontin production [90]. Again in models of immune-mediated liver injury, induced by alpha-galactosylceramide (alpha-GalCer) or Con A, FXR agonism reduced inflammation and simultaneously promoted the hepatic accumulation, function, and homing of immune-suppressive granulocytic myeloid–derived suppressor cells (MDSCs) [91].

#### Preclinical studies

FXR agonism has been proposed as a therapeutic intervention for cholestatic liver diseases because of its potential to prevent the accumulation of bile acids by regulating bile acid synthesis and transporters. During cholestasis, the expression of bile acid transporters is modulated to prevent the accumulation of bile acids in hepatocytes. This was evident in a mouse model of ANIT-induced cholestasis, where wild-type mice had a lower hepatic expression of the bile acid uptake transporter *Ostβ* and higher expression of the bile acid efflux transporter *Bsep*. *Fxr*^*−/−*^ mice lacked this response, suggesting FXR-dependency, and were more susceptible to liver injury [92]. Conversely, in rats with bile duct ligation and ANIT-induced cholestasis, FXR agonism decreased expression of bile acid synthesis genes and increased expression of genes related to canalicular bile acid transport such as *Bsep*, *Mrp2*, and *Mdr2*, which was associated with improved serum liver enzymes, markers of inflammation, liver damage, and decreased bile duct proliferation [93]. However, evidence for a positive role of FXR agonism in animal models of cholestasis is conflicting. *Bsep* upregulation by FXR agonism in a bile duct ligation model was also reported in another study; however, FXR agonism aggravated liver injury. Here, *Bsep* upregulation was regarded as counter-productive, as it would further promote bile duct obstruction by facilitating bile acid efflux from the hepatocyte [94]. Furthermore, another study reported *decreased* liver injury in bile duct–ligated *Fxr*^*−/−*^ mice [95].

#### Clinical studies

The FXR activators that have undergone or are undergoing clinical trial are the steroidal FXR agonist obeticholic acid (OCA) and the non-steroidal FXR agonists cilofexor, tropifexor, and MET409.

So far, numerous clinical trials for OCA in PBC patients with insufficient response to UDCA [96–102] have shown improvements in liver enzymes. Consequently, OCA is recommended as a second-line treatment in addition to UDCA for PBC patients with insufficient response to UDCA, and as a first-line treatment for patients who are intolerant to UDCA [103]. A large international placebo-controlled phase 4 trial is ongoing to further assess the effectiveness of OCA in PBC (NCT02308111), as well as a phase 3 study assessing OCA plus bezafibrate in PBC (NCT04594694). Mechanistically, OCA increased the transport of bile acids from hepatocytes to canaliculi in UDCA-treated PBC patients [104]. Importantly, the FDA advises against the prescription of OCA to PBC patients with advanced cirrhosis, as a result of 25 reports of liver decompensation or failure associated with OCA use. Most of these incidents involved patients with compensated cirrhosis, mostly with portal hypertension, or patients with decompensated cirrhosis. Liver decompensation occurred 10 days to 10 months after initiation of OCA [105]. In eligible patients, the recommended starting dose is 5 mg, which can be titrated to 10 mg after 3 months if OCA is well-tolerated. It is further recommended to monitor liver function before and after initiating OCA therapy [103].

In PSC, OCA brought a reduction in ALP in a phase 2 trial [106]; however, further clinical trials are not in the pipeline at this time [107]. OCA is also being tested in patients with pediatric biliary atresia (EudraCT 2014–004,693-42). Preliminary data showed that tropifexor improved γGT and ALT in PBC patients [108]. Cilofexor, another non-steroidal FXR agonist, decreased serum ALP, γGT, AST, ALT, and bile acid levels in PSC [109] and PBC patients, according to preliminary data [110]. A phase 3 clinical trial for cilofexor in PSC is underway (NCT03890120).

Regardless of the liver disease treated, the most common side effects of FXR agonists were dose-dependent pruritus, fatigue, and increased LDL:HDL ratio, which could increase atherosclerotic risk. The addition of statins to OCA mitigated the latter side effect [111]. Assessment of the interactions of antipruritus drugs with OCA is underway (NCT05133830). These side effects seem target-specific, as they occurred with different FXR agonists.

FXR modulation is a promising therapeutic avenue for various liver diseases, as highlighted by the approval of OCA for PBC and the numerous clinical trials in progress. However, the side effects of FXR agonists pose a challenge and the long-term efficacy still needs to be characterized. The working mechanisms of FXR modulation in the various liver diseases also need to be further elucidated.

### FGF19

Bile acid signaling is not restricted to the liver. Actually, a major part takes place in the intestine. An important hormone to fully understand the effects of (pan and intestinal) FXR activation by bile acids is FGF19, as it is mainly produced in the ileum in response to FXR activation by bile acids. After traveling from the ileum to the liver via the enterohepatic circulation, FGF19 exerts its effects through binding to the fibroblast growth factor receptor 4 (FGFR4) and its coreceptor β-klotho (KLB), which are mostly co-expressed in the liver [112].

In hepatocytes, FGF19 downregulates bile acid synthesis by inhibiting the major bile acid synthesis enzyme cholesterol 7-a-hydroxylase (CYP7A1) [113], a property that can be exploited to prevent bile acid overload-related liver injury. Because chronic FGF19 overexpression was shown to induce hepatocellular carcinoma in mice by activating STAT3 signaling [114, 115], a nontumorigenic FGF19 analogue (NGM282, also known as M70 and aldafermin) that does not activate STAT3 was developed [116]. NGM282 suppressed bile acid synthesis in humans [116] and reduced serum levels of hydrophobic bile acids in patients with NASH and PSC [117]. In mouse models of cholestasis induced by bile duct ligation or ANIT, NGM282 decreased the bile acid pool size and diminished liver injury [118]. In *Mdr2*^*−/−*^ mice, FGF19 and NGM282 decreased liver injury, inflammation, and fibrosis [119]. A different FGF19 analogue lacking tumorigenic properties, FGF19-M52, similarly protected *Mdr2*^*−/−*^ mice from cholestatic injury [120]. Moreover, constitutive activation of intestinal FXR in mice reduced the bile acid pool size and attenuated cholestatic injury caused by *Mdr2* deficiency, bile duct ligation, and ANIT treatment [121]. Consequently, NGM282 was tested in humans for the treatment of cholestatic disorders. In PBC patients with inadequate response to UDCA, NGM282 decreased ALP, GGT, ALT, AST, and IgM levels after 28 days of treatment. Diarrhea was the most commonly reported side effect [122]. In PSC patients, NGM282 treatment for 12 weeks improved fibrosis biomarkers; however, it did not improve ALP levels, the primary endpoint [123]. NGM is not planning to pursue further clinical trials of NGM282 for PSC and PBC at this time [124].

### TGR5

TGR5 (GPBAR1) is a G-protein-coupled receptor that is ubiquitously expressed. The highest mRNA expression of GPBAR1 is found in the gallbladder and in monocytes [56, 125]. In the liver, GPBAR1 is expressed by cholangiocytes, both intrahepatic [126] and extrahepatic [127], Kupffer cells [128], sinusoidal endothelial cells [129], activated hepatic stem cells [130], NTK cells [131], and hepatocytes [132]. In the intestine, GPBAR1 is mostly expressed by intestinal endocrine cells in the colon and goblet and Paneth cells in the small intestine [56, 133].

Known endogenous TGR5 ligands are bile acids, with potency for TGR5 activation LCA > DCA > CDCA > CA [134, 135]. Therefore, secondary bile acids produced by the gut microbiota are the preferred ligands for TGR5. Because TGR5 is expressed at the plasma membrane, unlike FXR, which is a nuclear receptor, TGR5 activation does not require bile acid entry into the cell. TGR5 can regulate various signaling pathways such as NF-κB, AKT, and ERK, among others [136]. TGR5 is also well-known as a regulator of energy and glucose metabolism [137].

As mentioned above, TGR5 is expressed in cholangiocytes, where it can be localized in the primary cilium, at the apical membrane and in intracellular vesicles, and its stimulation can cause opposite downstream effects depending on the subcellular location. TGR5 in cilia may come in contact with bile acids routinely, whereas TGR5 located on the apical membrane may be shielded from bile acids by a bicarbonate-rich apical glycocalyx [138]. *Tgr5*^*−/−*^ mice had decreased biliary proliferation in response to cholestasis and TGR5 agonists induced cholangiocyte proliferation [139]. Increased TGR5-mediated cell proliferation could potentially promote cholangiocarcinoma progression, also based on the observation that TGR5 is overexpressed in cholangiocarcinoma tissue [139, 140]. In gallbladder cholangiocytes, TGR5 colocalizes with cystic fibrosis transmembrane conductance regulator (CFTR) [127]. TGR5 activation increases intracellular cAMP levels and stimulates chloride secretion via CFTR [127], which could contribute to bicarbonate-rich fluid secretion [141]. Decreased bile flow in Tgr5^*−/−*^ mice and increased bile flow by TGR5 agonism were reported; however, it is not known whether these were CFTR-dependent [142]. Finally, TGR5 agonism increases cholangiocyte barrier function by stabilizing junctional adhesion molecule A (JAM-A) [143]. Although mutations leading to decreased TGR5 were identified in PSC patients, their rarity does not suggest these as a causative factor in PSC [144].

#### TGR5 and inflammation

**Fig. 4:**
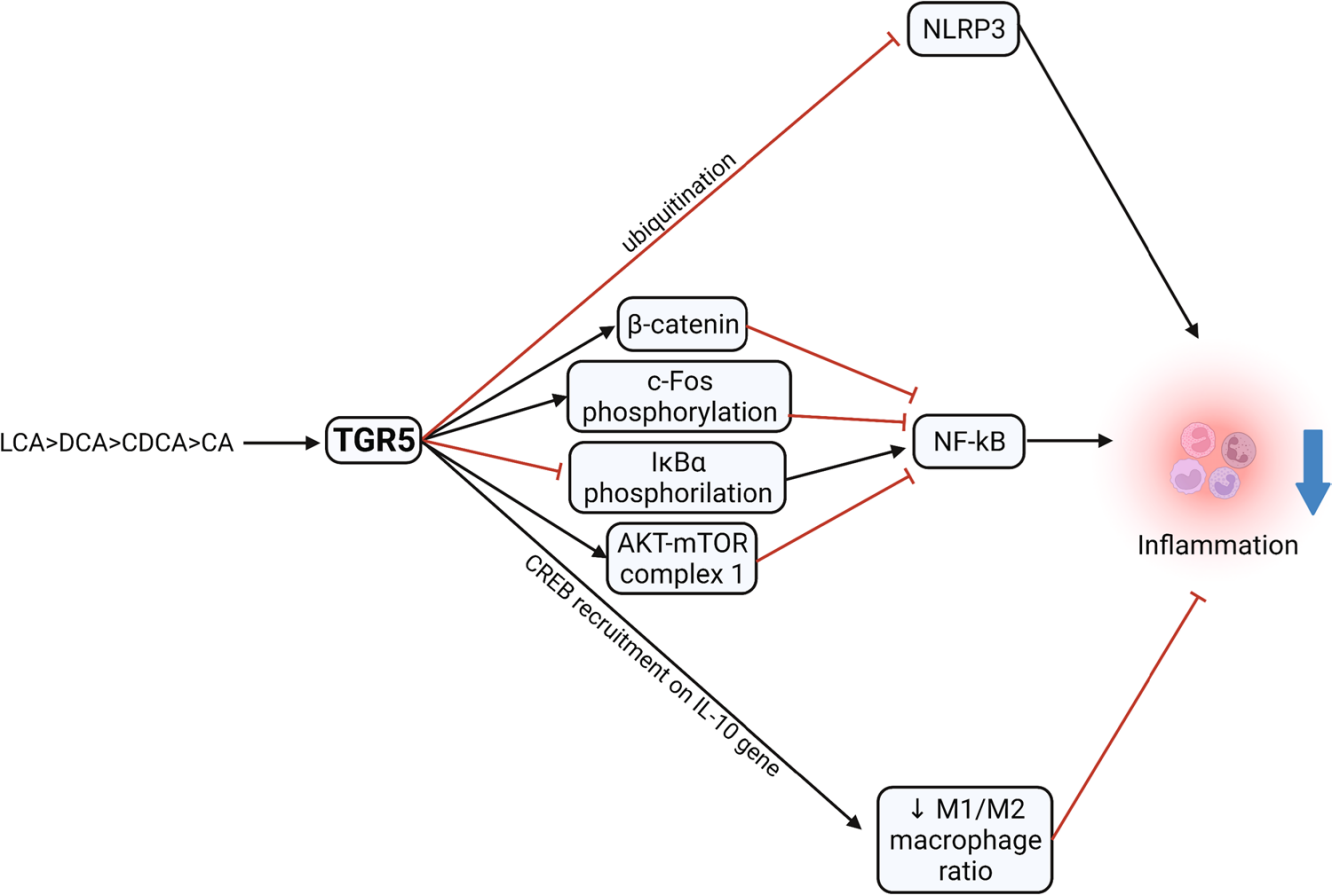
Effect of TGR5 activation on liver inflammation. Activation arrows are indicated in black and inhibition arrows in red. AKT-mTOR: protein kinase B—mammalian target of rapamycin signaling pathway; CA: cholic acid; CDCA: chenodeoxycholic acid; DCA: deoxycholic acid; IκBα: nuclear factor of kappa light polypeptide gene enhancer in B-cells inhibitor, alpha; LCA: lithocholic acid; NF-κB: nuclear factor kappa-light-chain-enhancer of activated B cells; NLRP3: NOD-, LRR- and pyrin domain–containing protein 3; TGR5: takeda-G-protein-receptor-5

TGR5 was first described as a bile acid receptor in monocytes and macrophages, where it suppressed their function in response to bile acids [134]. In macrophages, TGR5-mediated inhibition of cytokine production occurred through stabilization of the alternative (non-inflammatory) macrophage phenotype via CREB recruitment to the CRE on the promoter of the anti-inflammatory gene IL-10 [145–147]. TGR5 activation was further shown to downregulate cytokine production by inhibiting the NF-κB pathway in several cell types (Fig. 4), among which dendritic cells [148], NTK cells [131], endothelial cells [149], and macrophages and Kupffer cells [150–155]. In macrophages, NF-κB was inhibited by TGR5-dependent increased c-Fos phosphorylation [150], or by repressed phosphorylation of IκBα [151, 152]. Inhibition of macrophage chemokine production by TGR5 activation also occurred through activation of the AKT-mTOR complex 1, which promoted the translation of the C/EBPβ isoform LIP, which could blunt NF-κB activation [156]. In macrophages, TGR5 was also found to interfere with the β-catenin destruction complex to increase β-catenin levels, resulting in inhibition of the TLR4-NF-κB pathway via PI3K/Akt signaling [154] (Fig. 4).

Like FXR, TGR5 is involved in inflammasome regulation (Fig. 4). TGR5 activation by bile acids inhibited the NLRP3 inflammasome by increasing its ubiquitination, thereby reducing inflammation in vitro and in vivo [157–159]. In liver failure, raised serum bile acids correlate with infections and mortality. It was recently reported that the serum bile acid composition of patients with liver failure promotes TGR5 activation and reduces the pro-inflammatory response of monocyte in response to bacterial challenge. The patients with a TGR5-activating serum bile acid composition were at increased risk for a fatal outcome [160].

#### Preclinical studies

*Tgr5*^*−/−*^ mice are more susceptible to LPS-induced liver inflammation [151], which on the contrary is improved by TGR5 agonist treatment in wild-type mice [151]. *Tgr5*^*−/−*^ mice, which have a smaller, more hydrophobic bile acid pool [142], also exhibit more severe liver injury and inflammation after partial hepatectomy, which could be attenuated by cholestyramine treatment (a bile acid–binding resin that interrupts the enterohepatic circulation of bile acids) and Kupffer cell depletion [161].

*Tgr5*^*−/−*^ mice are also more susceptible to cholestatic damage caused by bile duct ligation, showing increased inflammatory cell infiltration [153, 154, 161], whereas TGR5 activation in wild-type mice protected against liver injury by decreasing the NF-κB pro-inflammatory response as well as oxidative stress [153]. Despite the positive effects of TGR5 activation in cholangiocytes detailed above, a TGR5 agonist alone did not improve the liver phenotype of *Mdr2*^*−/−*^ mice, and neither did an FXR agonist alone. However, dual TGR5/FXR agonist treatment reduced hepatic inflammation and fibrosis, probably by reducing bile acid synthesis in a FXR-dependent manner [162]. The lack of efficacy of the TGR5 agonist was likely due to the downregulation of TGR5 in the *Mdr2*^*−/−*^ mouse. TGR5 downregulation in biliary epithelial cells from *Mdr2*^*−/−*^ mice, a PSC model, was associated with a pro-inflammatory phenotype, which was reversed by TGR5 overexpression. TGR5 is also downregulated in the PSC liver [163].

In mouse models of acute immune-mediated hepatitis, *Tgr5*^*−/−*^ mice had a more severe liver injury, while a TGR5 agonist improved liver damage in wild-type mice by promoting a shift of NKT cells to a regulatory, IL-10 producing NKT cell subset [131].

In polycystic liver disease, antagonism rather than agonism of TGR5 could be beneficial. TGR5 was found to be overexpressed in cystic cholangiocytes, leading to increased cell proliferation and cyst growth. Deletion of *Tgr5* significantly reduced hepatic cystic areas in a mouse model of polycystic liver disease, whereas TGR5 agonists stimulated cyst growth in vitro [164, 165]. TGR5 antagonism may also be beneficial in cholangiocarcinoma. TGR5 expression is upregulated in cholangiocarcinoma and TGR5 agonism increased cell proliferation and migration in vitro and cholangiocarcinoma growth in vivo [140, 166].

#### Clinical studies

Given that TGR5 is expressed ubiquitously, systemic side effects are likely to occur. Although these were not studied in humans, mouse models provide hints as to which side effects may occur when modulating TGR5. TGR5 activation stimulated gallbladder filling and delayed emptying by promoting smooth muscle relaxation in mice [142], a side effect that could increase the risk of cholelithiasis and cholecystitis. TGR5 activation in endothelial cells increased the generation of the vasodilatory mediators nitric oxide [129, 149, 167] and hydrogen sulfide [168], while it inhibited the secretion of endothelin-1, a vasoconstrictor [169]. These effects may be exploited for reducing portal pressure, as demonstrated in a mouse model of hepatic cirrhosis [167]. However, they may result in peripheral arterial vasodilation, leading to blood pressure drops at therapeutic dosages, as reported in dogs, but not in rats [170]. Furthermore, in mice, overexpression of TGR5 in sensory nerves or activation by bile acids or TGR5 agonist administration induced pruritus and analgesia [171]. In light of the induction of cholangiocyte proliferation upon TGR5 activation [139], there is also a potential risk of cholangiocarcinoma development. TGR5 agonists that deactivate rapidly after exerting effects in the intestine were designed [170]; however, intestine-restricted action may not be desirable for the treatment of cholestatic and autoimmune liver diseases. The TGR5 agonist SB-756050 was studied in 51 type 2 diabetes patients. The compound was well-tolerated and there were no safety issues reported. A comprehensive description of side effects, however, is lacking [172].

Thanks to its anti-inflammatory properties, TGR5 *agonism* is an attractive treatment for autoimmune liver diseases, as shown by encouraging results in pre-clinical studies. Further studies are needed to characterize the levels of TGR5 expression in the context of liver diseases, which may determine the success of TGR5 agonism. TGR5 *antagonism,* rather than agonism, could be helpful in polycystic liver disease to reduce cyst growth and in cholangiocarcinoma to reduce cell proliferation and resistance to apoptosis. Unfortunately, clinical application of TGR5 modulators is hampered by the potential systemic side effects that stem from its ubiquitous expression, and translation of preclinical results to humans is thus largely undetermined.

### PXR

The pregnane-activated receptor (PXR, NR1I1) is a nuclear receptor highly and primarily expressed in intestinal enterocytes and liver hepatocytes [173]. PXR can be activated by numerous and structurally diverse ligands such as xenobiotics and natural and synthetic steroids, including the secondary bile acid lithocholic acid (LCA) [174]. PXR signaling is well-known to modulate the expression of drug-metabolizing enzymes and transporters (DMET) to facilitate xenobiotics metabolism, transport, and clearance, a function that is shared with the constitutive androstane receptor (CAR, NR1I3) [175, 176]. Besides DMET regulation, PXR is also involved in energy homeostasis [177], bile acid metabolism, and regulation of inflammation.

PXR is positively regulated by FXR [178] and the two receptors work synergistically to ensure bile acid homeostasis. Like FXR, PXR activation represses hepatic CYP7A1, the rate-limiting bile acid synthesis enzyme. PXR activation further promotes the expression of hepatocyte OATP2 (which can facilitate bile acid uptake by hepatocytes), CYP3A11 and SULT2A1 (which transform bile acids to promote their detoxification and excretion), and MRP2 (which promotes their canalicular transport) [174, 179–181]. These properties were hypothesized to counteract cholestatic injury. Accordingly, *Pxr*^*−/−*^ mice were more susceptible to LCA feeding [180] and cholestasis induced by bile duct ligation [182], and PXR agonist treatment reduced liver damage induced by both LCA and CA feeding [174, 180, 183] and by bile duct ligation [182] in wild-type mice.

Similar to other nuclear receptors, PXR expression is decreased by NF-κB activation, through interaction with RXRα, which heterodimerizes with PXR [184, 185]. A recent study showed that PXR can suppress both NF-κB and AP-1 signaling, thereby reducing the expression of inflammatory mediators. Accordingly, treatment with a PXR agonist repressed CCl_4_-induced expression of chemokine genes *Ccl2* and *Cxcl2* and reduced hepatic neutrophil infiltration and necrosis in mice [186]. Additionally, a role for PXR in the negative regulation of TLR4 in the intestine has emerged [187, 188].

However, there are potential adverse effects of PXR activation, specifically in metabolic health parameters. Studies in healthy volunteers showed that PXR activation by rifampin increased blood pressure, serum LDL, and total cholesterol, and worsened postprandial glucose tolerance [189–191]. Additionally, PXR was linked to chemoresistance in hepatocellular carcinoma by increasing the expression of DMET, as well as by inhibiting apoptosis [192]. Target-specific PXR activation that would circumvent adverse metabolic health effects, perhaps in combination with other treatments, could be useful for the treatment of liver diseases, however much remains to be explored.

## Bile acid–related treatments in cholestatic liver diseases

For cholestatic liver diseases, bile acid–related treatments aim at (a) reducing hepatic bile acid accumulation, (b) reducing bile acid toxicity, (c) promoting bile flow, and (d) reducing inflammation. As for (a) reducing hepatic bile acid accumulation, an approach is to reduce the bile acid pool size by interrupting the bile acid enterohepatic circulation (Fig. 1). This can be achieved by bile acid–binding resins (e.g., cholestyramine, which is used for cholestatic pruritus and hypercholesterolemia) or by inhibition of intestinal ASBT, as these treatments increase the fecal loss of bile acids. This treatment strategy is similar to partial external biliary diversion performed in children with PFIC and Alagille disease [193]. ASBT inhibitors improved cholestatic injury in mice [194, 195] and cholestatic pruritus in PBC patients [196]. The ASBT inhibitor maralixibat was recently approved for the treatment of cholestatic pruritus in patients with Alagille syndrome [197], whereas trials for other cholestatic disorders are ongoing [198, 199]. The most common side effects were diarrhea and abdominal pain. The deficiency of fat-soluble vitamins, which require bile acids for intestinal absorption, was also reported [196, 197]. Besides bile acid–binding resins and ASBT inhibition, inhibition of hepatic NTCP may reduce bile acid uptake by hepatocytes. Because hepatocyte NTCP is the entry receptor for hepatitis D virus (HDV), the NTCP inhibitor bulevirtide was recently approved in Europe for the treatment of chronic HDV infection in HDV RNA positive patients with compensated liver disease. In mouse models of cholestatic liver damage, bulevirtide attenuated liver injury by reducing biliary bile acid output and increasing biliary lipid output [200, 201]. Reduction of hepatic synthesis of bile acids can be achieved by agonists of FXR and PXR and recombinant FGF19, as discussed in the previous sections (Fig. 1). The FXR agonist OCA is approved for PBC patients, as discussed above. As for (b) reducing bile acid toxicity and (c) promoting bile flow, biliary bile acid composition can be modulated by UDCA, TUDCA, and norUDCA, which render bile less hydrophobic and thus less cytotoxic, and by NTCP inhibition, which increases the phospholipids/bile acid ratio [200, 201] (Fig. 1). (T)UDCA and norUDCA further promote bicarbonate-rich bile flow [202]. In particular, norUDCA escapes hepatic conjugation and can be thereby reabsorbed passively by the biliary epithelium, to be returned to hepatocytes for re-secretion (cholehepatic shunting). Both re-secretion by hepatocytes and bicarbonate secretion into bile upon norUDCA reabsorption can contribute to increased bile flow [203]. UDCA is approved for PBC, cholestasis of pregnancy, and cholesterol gallstone dissolution. UDCA is also used for PFIC3, cystic fibrosis–related liver disease (CFLD), and PSC, although long-term efficacy is uncertain due to the lack of large clinical trials [204]. norUDCA as a treatment for PSC is being evaluated in a phase 3 study (NCT03872921) after promising results in a phase 2 study [205]. As for (d) reducing inflammation, besides anti-inflammatory and immune-modulatory agents [206], bile acid–related targets include hepatic FXR, PXR, and TGR5 agonists, as discussed in the previous sections. Interestingly, immunomodulatory effects of norUDCA were recently demonstrated in *Mdr2*^*−/−*^ mice and mice infected with non-cytolytic lymphocytic choriomeningitis virus (LCMV), a model of non-cholestatic liver injury. By modulating mTORC1 activity in CD8^+^ cells, norUDCA impaired the activation-induced metabolic reprogramming of CD8^+^ cells and significantly alleviated hepatic inflammation [207]. UDCA also has anti-inflammatory actions, reviewed elsewhere [204]. In addition to cholestatic diseases, targeting FXR, FGF19, and TGR5 signaling have also shown important therapeutic benefits for the treatment of NASH [208].

The most benefit from these treatments could likely be obtained by combining several approaches, depending on the type of cholestasis. Choosing the right treatment at the appropriate time is also important. It was proposed that drugs reducing bile acid synthesis are best used early, when the hepatic adaptations to cholestasis, which include suppression of bile acid synthesis, are not yet established [11].

Bile acids shape the gut microbiome by providing feeding substrate and by exerting antimicrobial activities. In turn, the gut microbiome shapes the bile acid pool composition by carrying out enzymatic activities (e.g., deconjugation and dehydroxylation) that modify primary bile acids, resulting in bile acids that have different affinities to bile acid receptors and can thus influence bile acid receptors signaling [209]. The gut-liver axis has been implicated in the pathophysiology of several liver diseases and the gut microbiota is altered in several liver diseases. Therefore, modulation of the gut microbiota is a potential therapeutic approach and can be achieved by dietary changes, prebiotics, probiotics, antibiotics, as well as by fecal microbiota transplantation and bacteriophages [210]. The relevance of the gut microbiota in liver diseases has been recently reviewed [211].

## Conclusion

Bile acids and their receptors modulate inflammation in the context of liver diseases. On the one hand, bile acid accumulation in cholestasis causes hepatocellular damage and is pro-inflammatory (Fig. 2). On the other hand, activation of bile acid receptors by bile acids exerts anti-inflammatory actions by repressing NF-κB signaling and the NLRP3 inflammasome, among other pathways (Figs. 3 and 4). Additional anti-inflammatory effects obtained by modulating bile acid receptors arise from their roles in regulating bile acid homeostasis, which have the potential to attenuate cholestasis, as observed in preclinical studies. Clinical studies lead to the approval of several bile acid receptor and bile acid–related treatments, such as obeticholic acid, UDCA, and the ASBT inhibitor maralixibat. A large number of clinical trials are ongoing, especially for FXR agonists and recombinant FGF19 (Fig. 1). However, the clinical use of bile acid receptors modulators and other bile acid–related treatments is hampered by their (potential, long-term) side effects, which stem from ubiquitous bile acid receptors expression, breadth of target signaling pathways, or both. This is a challenge especially for TGR5 modulators. The first steps to circumvent these challenges are underway, with the development of pathway-specific modulators. A combination treatment with nuclear receptor ligands and bile acids with different therapeutic effects may also be of interest. Another aspect that should be taken into account when designing clinical trials for cholestatic liver diseases is the anatomical heterogeneity of the disease process and the “ascending” pathogenesis of cholestatic liver diseases, as discussed by Jansen et al. [11]. Along with clinical trials, preclinical studies remain essential to further characterize the downstream effects of bile acid receptors modulation and to elucidate the working mechanisms in various liver diseases.

## Abbreviations

ANIT: Alpha-naphthylisothiocyanate
alpha-GalCer: Alpha-galactosylceramide
AP-1: Activator protein 1
ASBT: Apical sodium–dependent bile acid transporter
ASC: Apoptosis-associated speck-like protein containing a CARD
BSEP: Bile salt export pump
CA: Cholic acid
CAR: Constitutive androstane receptor
CDCA: Chenodeoxycholic acid
CCL2: C-C motif chemokine ligand 2
C/EBPβ: CCAAT/enhancer-binding protein β
CFTR: Cystic fibrosis transmembrane conductance regulator
CFLD: Cystic fibrosis–related liver disease
Con A: Concanavalin A
CRE: CAMP response element
CREB: CAMP response element-binding protein
CYP3A11: Cytochrome P450 3A11
CYP450: Cytochrome P450 family 7 subfamily A member 1
CYP7A1: Cholesterol 7-a-hydroxylase
Cxcl2: X-X-C motif chemokine ligand 2
DAMPs: Damage-associated molecular patterns
DCA: Deoxycholic acid
DMET: Drug metabolizing enzymes and transporters
FGF19: Fibroblast growth factor 19
FGFR4: Fibroblast growth factor receptor 4
FIC1: Familial intrahepatic cholestasis 1
FXR: Farnesoid X receptor
HSC: Hepatic stellate cells
HDV: Hepatitis D virus
IκBα: Nuclear factor of kappa light polypeptide gene enhancer in B-cells inhibitor, alpha
JAM-A: Junctional adhesion molecule A
KLB: Coreceptor β-klotho
LCA: Lithocholic acid
LIP: Liver-inhibitory protein
lncRNA H19: Long non-coding RNA H19
LPS: Lipopolysaccharide
MCA: Muricholic acid
MDR2: Multidrug resistance protein 2
MDR3: Multidrug resistance protein 3
MDSCs: Myeloid-derived suppressor cells
MRP2: Multidrug resistance–associated protein 2
mTOR complex 1: Mammalian target of rapamycin complex 1
NASH: Non-alcoholic steatohepatitis
NTCP: Na^+^-dependent taurocholate cotransporting peptide
NFAT: Nuclear factor of activated T-cells
NF-κB: Nuclear factor kappa-light-chain-enhancer of activated B cells
NLRP3: NOD-, LRR-, and pyrin domain–containing protein 3
norUDCA: Nor-ursodeoxycholic acid
NR1H4: Nuclear receptor subfamily 1 group H member 4
OATP2: Organic anion–transporting polypeptide 2
OCA: Obeticholic acid
PAMPs: Pathogen-associated molecular patterns
PBC: Primary biliary cholangitis
PFIC1: Progressive familial intrahepatic cholestasis type 1
PFIC2: Progressive familial intrahepatic cholestasis type 2
PFIC3: Progressive familial intrahepatic cholestasis type 2
PSC: Primary sclerosing cholangitis
PXR: Pregnane X receptor
SHP: Small heterodimer partner
SOCS3: Suppressor of cytokine signaling 3
STAT3: Signal transducer and activator of transcription 3
SULT2A1: Sulfotransferase family 2A member 1
TCA: Tauro-cholic acid
TGR5: Takeda-G-protein-receptor-5
TLR9: Toll-like receptor 9
TUDCA: Tauro-ursodeoxycholic acid
UDCA: Ursodeoxycholic acid
